# Efficacy and Safety of Placebo During the Maintenance Therapy of Ovarian Cancer in Randomized Controlled Trials: A Systematic Review and Meta-analysis

**DOI:** 10.3389/fonc.2022.796983

**Published:** 2022-05-25

**Authors:** Jin-feng Wang, Lan-bo Zhao, Ya-di Bin, Kai-lu Zhang, Chao Sun, Yi-ran Wang, Xue Feng, Jing Ji, Li-song He, Fang-yao Chen, Qi-ling Li

**Affiliations:** ^1^ Department of Obstetrics and Gynecology, First Affiliated Hospital of Xi’an Jiaotong University, Xi’an, China; ^2^ School of Finance, Xi’an Eurasia University, Xi’an, China; ^3^ Department of Epidemiology and Biostatistics, School of Public Health, Xi’an Jiaotong University Health Science Center, Xi’an, China

**Keywords:** ovarian cancer, maintenance therapy, efficacy, safety, placebo, randomized controlled trials

## Abstract

**Introduction:**

This meta-analysis evaluated the efficacy and safety of placebo during the maintenance therapy of ovarian cancer (OC) patients in randomized controlled trials (RCTs).

**Methods:**

A comprehensive literature review was performed for RCTs published up to and including August 2020 from four electronic databases. We analyzed the efficacy and safety in the control arms of the maintenance therapy in advanced OC patients. Hazard ratios (HRs) and the corresponding 95% confidence intervals (CIs) of progression-free survival (PFS) and overall survival (OS) were estimated in the placebo arms and the observation arms, respectively, using the Frequency Framework method. We also calculated the incidences of common adverse effects (AEs) in the placebo arms.

**Results:**

In total, 41 articles with 20,099 (4,787 in the placebo arms, 3,420 in the observation arms, and 11,892 in the experiment arms) patients were included in this meta-analysis. Compared with observation, placebo did not improve or reduce PFS (HR, 1.02; 95% CI, 0.87–1.20; *P* = 0.81) and OS (HR, 1.02; 95% CI, 0.89–1.16; *P* = 0.76) of OC patients, while other treatments, except for radiotherapy, significantly improved PFS and OS (all *P* < 0.05). The incidences of AEs produced by placebo were 94.03% in all grades and 20.22% in grade ≥3. The incidences of AEs were 29.75% in fatigue, 26.38% in nausea, 24.34% in abdominal pain, 18.92% in constipation, 16.65% in diarrhea, 14.55% in vomiting, 13.89% in hypertension, and 13.14% in headache.

**Conclusions:**

Placebo did not improve or reduce the PFS and OS benefits of OC patients in RCTs but increased the incidences of AEs.

## Introduction

Ovarian cancer (OC) is one of the most common malignant reproductive tumors in women, with high recurrence rate and high mortality. Its median progression-free survival (PFS) and median overall survival (OS) range from 16 to 21 months and from 24 to 60 months, respectively (1). OC is the main cause of death among women in the USA and worldwide, accounting for the fifth and eighth, respectively (2, 3). In 2021, a total of 21,410 newly diagnosed cases and 13,770 deaths due to OC were estimated in America (2).

The standard first-line therapy of OC is debulking surgery in combination with chemotherapy based on paclitaxel and platinum, which can relieve the symptoms of the patients and achieve no evidence of disease progression temporarily. About 70% of the patients encounter a recurrence within 3 years (4). Therefore, many researchers put forward maintenance therapy, including extra-chemotherapy, immunotherapy, antiangiogenic inhibitors (AIs), selective small-module (SSM) inhibitors, and poly-ADP-ribose polymerase (PARP) inhibitors (4–8). All of them were added following complete or partial remission of chemotherapy in order to eschew disease progression and increase PFS and OS. Many randomized controlled trials (RCTs) further confirmed the efficacy and the safety of these maintenance therapies (4, 9–11). Some maintenance drugs, such as olaparib and niraparib, have shown significant positive effects on PFS and OS (4, 9). Most clinical trials on immunotherapy (7, 12–15), chemotherapy (6, 16–18), and SSM inhibitors (8) exhibited negative effects on PFS and OS, with a few studies gaining opposite results (16, 19). However, whether the control arms of RCTs, including placebo or observation, basically have effects on survival in the maintenance process is undefined. Until now, scarce reports have been published on the positive effects of placebo in RCTs.

Placebo, with a long history, mainly contains three forms: pharmacologic (a tablet), physical (a manipulation), and psychological (a conversation) (20). Recently, placebo tablets, an inert substance, have been administrated blindly to patients in clinical trials with an expectation for such to produce clinical benefits through the interaction with a caregiver and healthcare systems (21). The positive effects produced by placebo in clinical trials are not affected by its pharmacologic or physiologic properties (22). The positive effects are the evolution of the disease process altered in a positive direction (22). Researchers compared the differences between placebo and observation directly in the aspects of subjective and objective outcomes, including pain, psychopathy, hypertension, and so on (20). They found that placebo exhibited a few benefits on continuous subjective outcomes and the treatment of pain, but it did not have effects on objective or binary outcomes. Jonas *et al.* found that the placebo effect was related to the size, color, and label of tablets (23). I Požgain *et al.* showed that placebo in RCTs was effective for the health status of patients because of their own beliefs (24). Julia W. Haas *et al.* concerned about the effect of blindness caused by placebo on the treatment of patients with irritable bowel syndrome. The results found that patients with double-blind placebo possessed more enthusiasm; however, those with open-label placebo were contradicted, attributing the improvement of symptoms more to psychological function instead of the treatment itself (25). About 25% patients (26) and 19% healthy volunteers (27) taking a placebo experienced adverse events (AEs). Maxine de la Cruz *et al.* found that there was a placebo response in clinical trials about the treatment of fatigue in advanced cancer patients (28). However, no comparative study has been established to prove the differences between placebo and observation in the efficacy and AEs of the maintenance therapy of OC RCTs yet.

In this meta-analysis, we mainly compared the differences of median PFS and median OS between patients in the placebo and observation arms and illustrated the safety of placebo in RCTs with the maintenance therapy of advanced OC.

## Patients and Methods

### Search Strategy

According to the Preferred Reporting Items for Systematic Review and Meta-analysis (PRISMA) guidelines (29), electronic databases (PubMed, Web of Science, Embase, and Cochrane Library) were searched from their inception to August 2020 to obtain relevant RCTs. The search terms included “ovarian cancer” or “ovarian neoplasms”, “placebo” or “maintenance” or “consolidation” or “observation” or “natural history”, and “randomized controlled trial”. We also performed a manual search to find potential relative RCTs by using the reference lists of key articles. The language of all RCTs was limited to English.

### Inclusion and Exclusion Criteria

The inclusion criteria were as follows: (1) population: patients with FIGO stage IIB–IV epithelial ovarian, fallopian tube, primary peritoneal cancer or platinum-sensitive recurrent ovarian cancer (ROC) who are receiving maintenance therapy; (2) study design and comparators: phase II or III RCTs with control arms, including placebo or observation; (3) interventions: no other anticancer treatments except for standard front-line chemotherapy added to placebo or observation arms; (4) outcomes: mature data of median PFS or median OS reported as hazard ratios (HRs) and 95% confidence intervals (CIs), the number of patients with common AEs in the placebo arm, and the total number of patients receiving placebo; and (5) the latest articles were applied when duplicate publications existed or when publications were continuously updated.

The exclusion criteria were as follows: (1) not RCTs, including abstract, meeting, case, editorial, review, and so on; (2) platinum-resistant advanced OC or ROC; (3) animal trials; (4) other antitumor agents in the control arms; (5) no long-term outcomes covered in the study; and (6) unavailable research data.

No ethical approval and patient consent were required because the meta-analysis was performed based on previously published studies.

### Outcome Measures

The primary outcome of this meta-analysis was median PFS. Median OS and AEs were the secondary outcomes. The AEs were in accordance with the National Cancer Institute Common Terminology Criteria for Adverse Events, version 4.0.

### Assessment of the Risk of Bias and Data Extraction

We assessed the potential risk of bias in the trials using the Cochrane Collaboration Risk of Bias Assessment tool, which involved the following domains: (1) random sequence generation (selection bias); (2) allocation concealment (selection bias); (3) blinding of participants, personnel, and outcome assessment (performance bias and detection bias); (4) incomplete outcome data (attrition bias); and (5) selective reporting data (reporting bias) (30). The risks were divided into three levels: high, unclear, and low. Two reviewers (Wang and Sun) completed the review independently. Disagreements were resolved by a discussion.

Two reviewers (Wang and Sun) independently extracted the baseline information from each study, including data on author, year of publication, RCT phase, and the number of experimental arms and control arms. The primary and secondary endpoints included median PFS, median OS, the corresponding HR and 95% CIs, and the number of common AEs.

### Statistical Analysis

We indirectly compared the median PFS and median OS between the placebo arms and the observation arms by using HR and 95% CIs in view of the Frequency Framework method. We calculated the corresponding HR and 95% CIs by combining the HR with the 95% CIs of all subgroups in view of the generic inverse of variance method with a fixed-effect model (31). The incidences of common AEs were calculated by IBM SPSS Statistics (version 20.0) using the number of patients with common AEs in the placebo arms and the total number of patients receiving placebo. Heterogeneity among the studies was evaluated by the inconsistency index (*I* ^2^) value. If *I* ^2^ ≥ 50% or *P < *0.1, a random-effect model was used to reduce the heterogeneity and increase the reliability; if *I* ^2^ < 50% or *P >* 0.1, a fixed-effect model was used. All statistical tests were two-sided, *P* < 0.05 was considered statistically significant (22). We used Review Manager (version 5.3, the Cochrane library) for the assessment of the risk of bias and R software (version 3.4.4, the R Foundation for Statistical Computing) for network meta-analyses.

## Results

### Study Selection and Characteristics

**Figure 1:**
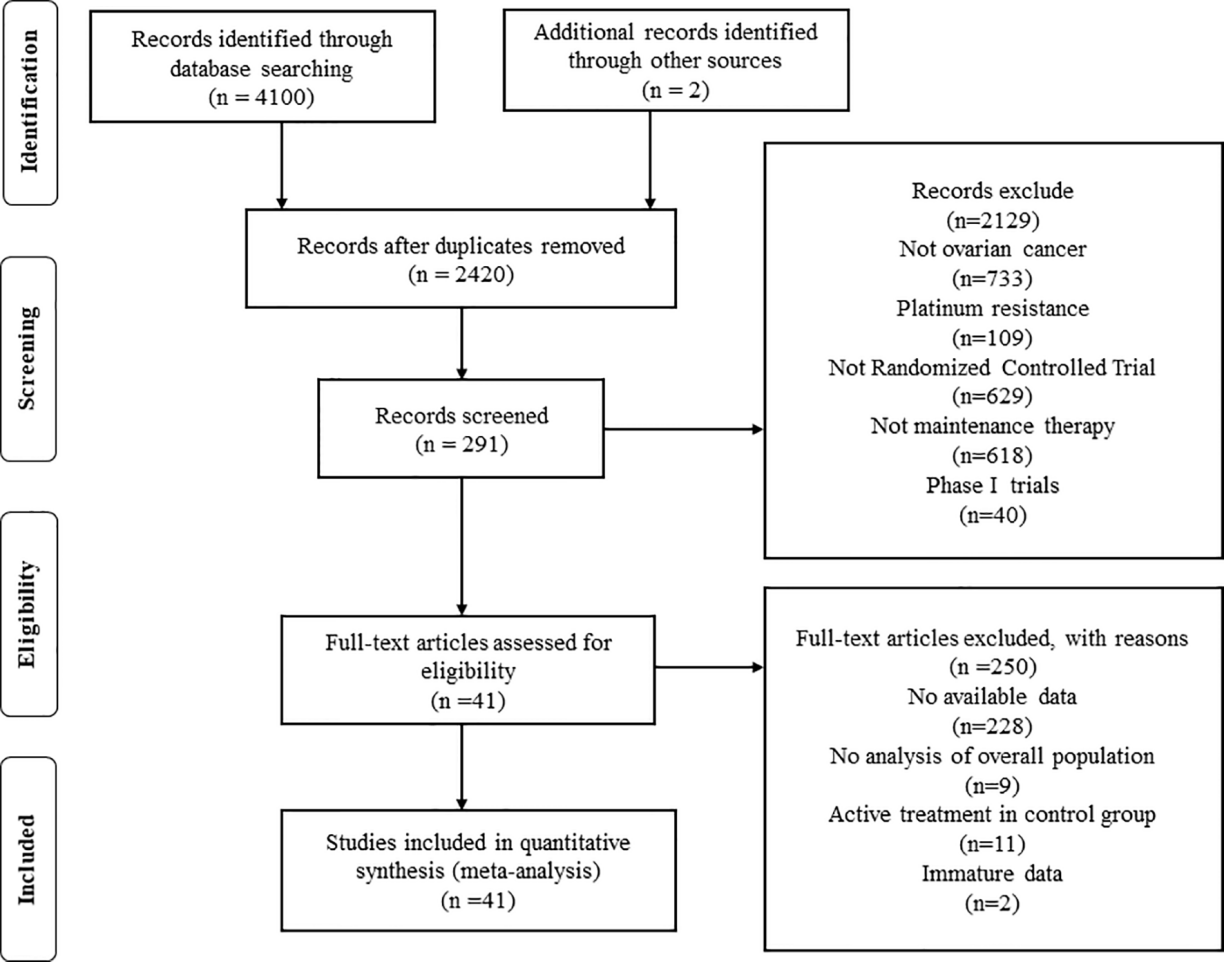
Preferred Reporting Items for Systematic Review and Meta-analysis flow chart of the study selection process.

**Figure 2:**
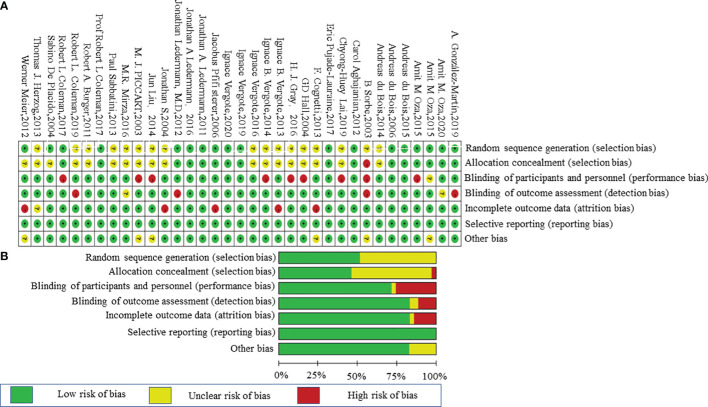
Risk-of-bias graph. **(A)** Review authors’ judgments about each risk-of-bias item presented as percentages across all included studies. **(B)** Review of authors’ judgments about each risk-of-bias item for each included study.

**Figure 3:**
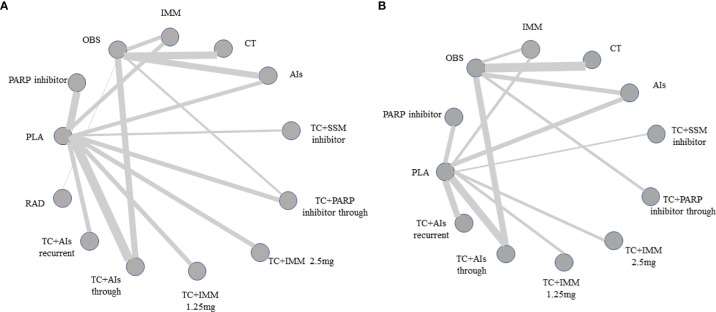
Network of treatment comparisons for overall efficacy. **(A)** Network plot of treatment comparisons of progression-free survival. **(B)** Network of treatment comparisons of overall survival. Directly comparable treatments are linked with a line, the thickness of which corresponds to the number of trials that assessed the comparison. AIs, angiogenesis inhibitors; PLA, placebo; TC, platinum plus paclitaxel; CT, chemotherapy; OBS, observation; PARP inhibitors, poly (ADP-ribose) polymerase inhibitors; IMM, immunotherapy; RAD, radiotherapy; SSM inhibitors, selective small-module inhibitors.

A total of 4,102 studies were retrieved through searching the electronic databases and other sources. Forty-one studies with 20,099 patients (experiment arms = 11,892; placebo arms = 4,787; observation arms = 3,420) that met the inclusion criteria were retained for comparison analysis. The PRISMA flow chart summarizing the process of evidence acquisition is shown in **Figure 1**. The flow chart mapped out the number of studies identified, screened, included, and excluded as well as the reasons for exclusions. The included studies were published between 2003 and 2020. The control arms of these RCTs consisted of 21 placebos (10 placebo maintenance after chemotherapy and 11 placebo through maintenance with and after chemotherapy) and 14 observations. Among 21 placebo studies (1, 5, 8, 9, 11, 12, 14, 15, 32–49), which all analyzed mature median PFS, 15 studies calculated the median OS, with 9 results being mature. There were 14 studies in the observation arms (6, 7, 13, 16–19, 50–57), all of which analyzed the mature median PFS and 13 studies analyzed the median OS, with 9 studies acquiring mature results. One study with secondary cytoreduction in the observation arms (57), one study with only a subgroup analysis (40), and one phase Ib/II study after extracting phase II data (8) were included in this meta-analysis. Except for one study (8), the other placebo arms with 2,665 patients all reported AEs in detail. The main characteristics and outcomes of the included RCTs are summarized in **Table 1**. **Figure 2** shows the results about the assessment of the risk of bias in the included RCTs according to the Cochrane Collaboration Risk of Bias Assessment tool. The network plots of the direct comparisons in the maintenance therapy of OC for PFS and OS are displayed in **Figures 3A, B**.

**Table 1:** Baseline characteristics of the included studies.

Author	Phase	Treatment	Number	Primary endpoint		Second endpoint	Median progressive-free survival (months)	Hazard ratio (HR)	95% CI	Median overall survival (months)	HR	95% CI
Placebo arm in the control arm												
Jonathan S, 2004 (12)		Oregovomab	73		PFS		13.3	0.927	0.621–1.383			
		Placebo	72				10.3					
Paul Sabbatini, 2013, MIMOSA Study (14)	III	Abagovomab	593		PFS	OS	13.43	1.099	0.919–1.315	NR	1.15	0.872–1.518
		Placebo	295				13.4			NR		
Thomas J. Herzog, 2013 (11)	II	Sorafenib	123		PFS	OS	12.7	1.09	0.72–1.63	NR	1.49	0.69–3.23
		Placebo	123				15.7			NR		
Andreas du Bois/I. Vergote, 2014/2019, AGO-OVAR16 (38, 43)	III	Pazopanib	472		PFS	OS	17.9	0.77	0.64–0.91	59.1	0.96	0.805–1.145
		Placebo	468				12.3			64		
		Placebo	131				13.8			3 years OS rate: 80%		
A. González-Martín, 2019, PRIMA/ENGOT-OV26/GOG-3012 (9)	III	Niraparib	487		PFS	OS	13.8	0.62	0.5–0.76	2 years OS rate: 84%	0.7	0.44–1.11
		Placebo	246				8.2			2 years OS rate: 77%		
Robert A. Burger/Krishnansu S. Tewari, 2011/2019, GOG-0218 (35, 37)	III	(TC + bevacizumab) + bevacizumab	623		PFS	OS	14.1	0.717	0.625–0.824	43.4	0.96	0.85–1.09
		bevacizumab initiation treatment	625				11.2	0.908	0.795–1.040	40.8	1.06	0.94–1.2
		(TC + placebo) + placebo	625				10.3			41.1		
Ignace B. Vergote, 2013 (1)	II	(TC + enzastaurin) + enzastaurin	69		PFS		18.9	0.8	0.5–1.29			
		(TC + placebo) + placebo	73				15.2					
Andreas du Bois/Isabelle Ray-Coquard, 2015/2019, AGO-OVAR 12 (36)	III	(TC + nintedanib) + nintedanib	911		PFS	OS	17.6	0.86	0.75–0.98	62	0.99	0.83–1.17
		(TC + placebo) + placebo	455				16.6			62.8		
Ignace Vergote, 2019, TRINOVA-3/ENGOT-ov2/GOG-3001 (48)	III	(TC + trebananib) + trebananib	678		PFS		15.9	0.93	0.79–1.09	46.6	0.99	0.79–1.25
		(TC + placebo) + placebo	337				15			43.6		
Robert L. Coleman, 2019 (49)	III	(TC + veliparib) + veliparib	382		PFS	OS	23.5	0.68	0.56–0.83	NE		
		Veliparib combination only	383							NE		
		(TC + placebo) + placebo	375				17.3			NE		
Jonathan A. Ledermann, 2011 (45)	II	BIBF 1120	43		PFS	OS	36-week PFS rate: 16.3%	0.65	0.42–1.02	NE	0.84	0.51–1.39
		Placebo	40				36-week PFS rate: 5%			NE		
Jonathan Ledermann, MD/Michael Friedlander, 2012/2018 (39)	II	Olaparib	136		PFS		8.4	0.35	0.25–0.49	29.8	0.73	0.55–0.95
		Placebo	129				4.8			27.8		
M.R. Mirza, 2016, ENGOT-OV16/NOVA trial (40)	III											
gBRCA cohort		Niraparib	138		PFS		21	0.27	0.17–0.41			
		Placebo	65				5.5					
Non-gBRCA cohort		Niraparib	234				9.3	0.45	0.34–0.61			
		Placebo	116				3.9					
Eric Pujade-Lauraine, 2017, SOLO2/ENGOT-Ov21 (42)	III	Olaparib	196		PFS	OS	19.1	0.3	0.22–0.41	NR	0.8	0.3–1.31
		Placebo	99				5.5			NR		
Prof. Robert L. Coleman, 2017, ARIEL3 (46)	III	Rucaparib	375		PFS		10.8	0.36	0.3–0.45			
		Placebo	189				5.4					
Carol Aghajanian, 2012/2015, OCEANS (5, 34)	III	(GC + bevacizumab) + bevacizumab	242		PFS	OS	12.4	0.484	0.388–0.605	33.6	0.95	0.77–1.18
		(GC + placebo) + placebo	242				8.4			32.9		
F. Cognetti, 2013, AGO-OVAR 2.14 (44)	II	(TC + zibotentan) + zibotentan	59		PFS	OS	7.6	1.46	80% CI: 1.1–1.94			
		(GC + placebo) + placebo	61				10					
Ignace Vergote, 2016 (15)	III	(TC + farletuzumab, 1.25 mg/kg) + farletuzumab, 1.25 mg/kg	370		PFS	OS	9.5	0.99	0.81–1.21	28.7	0.99	0.78–1.27
		(TC+ farletuzumab, 2.5 mg/kg) Farletuzumab, 2.5mg/kg	366				9.7	0.86	0.7–1.06	32.1	0.88	0.68–1.13
		(TC + placebo) + placebo	364				9			29.1		
Jonathan A. Ledermann, 2016, ICON6 (33)	III	(TC + cediranib) + cediranib	164		PFS	OS	11	0.56	0.44–0.72	26.3	0.77	0.55–1.07
		(TC + cediranib) + placebo	174				9.9	NR	NR			
		(TC + placebo) + placebo	118				8.7			21		
Ignace Vergote, 2020 (8)	Ib/II	(GC + ralimetinib) + ralimetinib	58		PFS	OS	10.25	0.773	90% CI: 0.535–1.117	29.17	0.83	90% CI: 0.538–1.27
		(GC + placebo) + placebo	52				7.92			25.1		
Amit M. Oza, 2020 (32)	II	(TC + adavosertib) + adavosertib	59		PFS	OS	9.9	0.55	0.32–0.95	NR	1	0.53–1.88
		(TC + placebo) + placebo	62				8			35.4		
Observation arm in the control arm												
B. Sorbe, 2003 (54)		Chemotherapy	35		PFS	OS	5 years: 36%; 37	0.72	0.4–1.3			
		Radiotherapy	32				5 years: 56%; 116	0.52	0.27–0.99		5 years: 69%	
		Observation	31				5 years: 35%; 32					
M.J. Piccart, 2003 (16)	III	Cisplatin	76		OS	PFS	4.625	0.89	0.59–1.33	6.96	0.82	0.52–1.29
		Observation	76				3.45			5.875		
Sabino De Placido, 2004 (6)	III	Topotecan	137		PFS	OS	18.2	1.18	0.86–1.63			
		Observation	136				28.4					
G.D. Hall, 2004 (7)	III	Interferon-alpha (INFa) 2a	149		PFS, OS		10.3	0.96	0.75–1.22	27	1.06	0.82–1.38
		Observation	149				10.4			32.7		
Ignace B. Vergote, 2014 (55)	III	Erlotinib	420		PFS	OS	12.7	1.05	0.9–1.23	50.8	0.99	0.81–1.2
		Observation	415				12.4			59.1		
Jun Liu, 2014 (50)		Autologous cytokine-induced killer cells	46		PFS	OS	37.7	0.493	0.302–0.807	61.5		
		Observation	46				22.2			55.9		
H.J. Gray, 2016 (13)	II	Cvac	29		PFS	OS	13	0.72	0.38–1.38	NR	0.38	Not shown
		Observation	27				9			NR		
Chyong-Huey Lai, 2019 (19)	III	Pegylated liposomal doxorubicin/carboplatin	23		PFS	OS	55.5	0.4	0.19–0.87	NR	0.53	0.22–1.27
		Observation	21				9.2			95.1		
Andreas du Bois, 2006 (17)	III	(TC + epirubicin) + epirubicin	647		OS	PFS	18.4	0.95	0.83–1.07	45.8	0.93	0.81–1.08
		Observation	635				17.9			41		
Jacobus Pfifi Sterer, 2006 (18)	III	TC + topotecan	658		OS	PFS	18.2	0.97	0.85–1.1	43.1	1.01	0.86–1.18
		Observation	650				18.5			44.5		
Werner Meier, 2012 (51)	II	(TC + lonafarnib) + lonafarnib	53		NR	NR	14.2	0.78	0.5–1.2	34.4	0.62	0.4–1.1
		Observation	52				17.8			47.3		
Amit M. Oza/Timothy J. Perren, 2015, ICON7 (52)	III	(TC + bevacizumab) + bevacizumab	764		PFS	OS	19.8	0.87	0.77–0.99	58	0.99	0.85–1.14
		Observation	764				17.4			58.6		
Amit M. Oza, 2015 (53)	II	(TC + olaparib) + olaparib	81		PFS	OS	12.2	0.51	0.34–0.77	33.8	1.17	0.79–1.73
		Observation	81				9.6			37.6		
Robert L. Coleman, 2017, NRG Oncology/Gynecologic Oncology Group study GOG-0213 (57)	III	(TC + bevacizumab) + bevacizumab	337		OS	PFS	13.8	0.628	0.534–0.739	42.2	0.83	0.683–1.005
		Observation	337				10.4			37.3		

TC, paclitaxel and carboplatin; GC, gemcitabine and carboplatin; PFS, progression-free survival; OS, overall survival; HR, hazard ratio; 95% CI, 95% confidence interval.

### Progression-Free Survival

**Figure 4:**
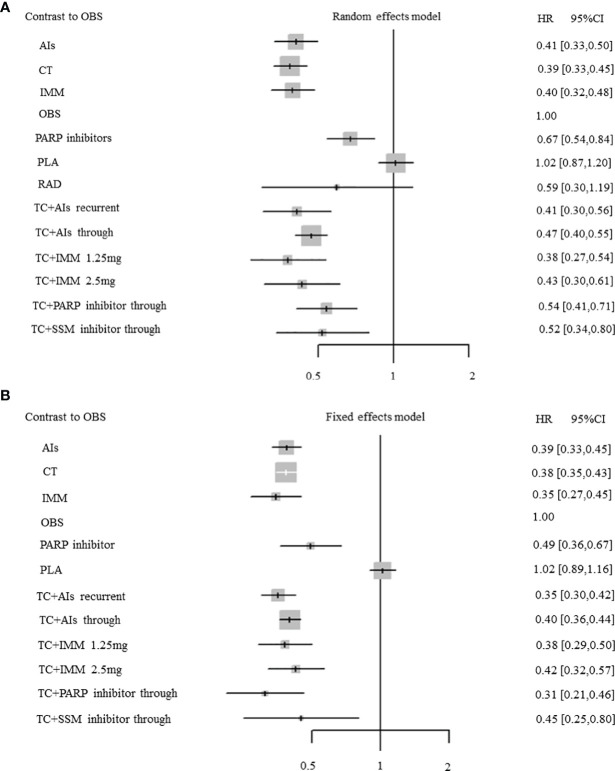
Forest plots. **(A)** Progression-free survival (PFS). **(B)** Overall survival. Hazard ratios and 95% confidence intervals (95% CIs) of each treatment *versus* observation in the maintenance therapy of ovarian cancer. Central dots represent medians; lines represent 95% CIs.

**Figure 5:**
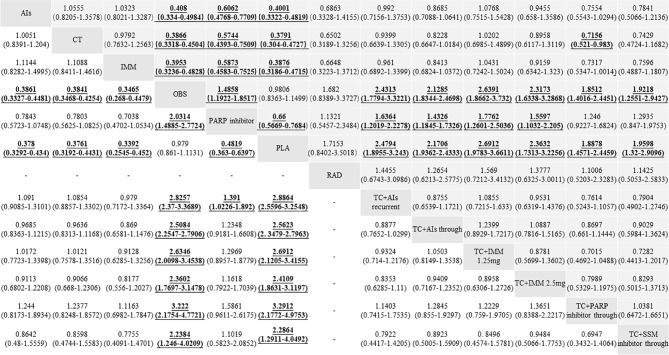
Hazard ratios (HRs) and 95% confidence intervals (95% CIs) of efficacy among the maintenance therapies of ovarian cancer patients between progression-free survival (PFS, up) and overall survival (OS, down). Comparisons between treatments should be read from left to right, and the estimate is in the cell in common between the column-defining treatment and the row-defining treatment. For efficacy, HR lower than 1 and 95% CI not including 1 favor the row-defining treatment of PFS or the column-defining treatment of OS. To obtain the HRs for comparisons in the opposite direction, reciprocals should be taken. Significant results are in bold and underlined.

A total of 35 RCTs (4 RCTs: AIs with 1,058 patients; 10 RCTs: TC + AIs through with 3,990 patients; 1 RCT: TC + AIs current with 625 patients; 5 RCTs: PARPi with 1,566 patients; 2 RCTs: TC + PARPi through with 463 patients; 5 RCTs: immunotherapy with 890 patients; 1 RCT: TC + immunotherapy at 1.25 mg with 370 patients; 1 RCT: TC + immunotherapy at 2.5 mg with 366 patients; 6 RCTs: chemotherapy with 1,576 patients; 1 RCT: radiotherapy with 32 patients; 2 RCTs: TC + selective small-molecule inhibitor through with 117 patients; 21 RCTs: placebo with 4,606 patients; and 14 RCTs: observation with 3,420 patients) were included, which reported mature data on the PFS of OC patients with maintenance therapy. There was significant heterogeneity among RCTs (overall: *I* ^2^ = 49.6%; *P* = 0.002), so the pooled HR was calculated by using a random-effect model. Except for placebo (HR, 1.02; 95% CI, 0.87–1.20; *P* = 0.81) and radiotherapy (HR, 0.59; 95% CI, 0.30–1.19; *P* = 0.13), other treatments significantly improved the PFS when compared with observation (all *P <*0.05) (**Figure 4A**). Compared with PARP inhibitors indirectly, PFS was significantly improved in AIs (HR, 0.61; 95% CI, 0.48–0.77; *P* < 0.001), chemotherapy (HR, 0.57; 95% CI, 0.44–0.75; *P* < 0.001), immunotherapy (HR, 0.59; 95% CI, 0.46–0.75; *P* < 0.001), chemotherapy combined with AIs recurrent (HR, 0.61; 95% CI, 0.45–0.83; *P* < 0.002), chemotherapy combined with AIs through (HR, 0.70; 95% CI, 0.58–0.84; *P* < 0.001), chemotherapy combined with immunotherapy at 1.25 mg (HR, 0.56; 95% CI, 0.40–0.79; *P* < 0.001), and chemotherapy combined with immunotherapy at 2.5 mg (HR, 0.64; 95% CI, 0.45–0.91; *P* = 0.012) (**Figure 5**).

### Overall Survival

Among 28 RCTs about median OS, 18 studies (3 RCTs: AIs with 935 patients; 7 RCTs: TC+AIs through with 2,861 patients; 1 RCT: TC + AIs recurrent with 625 patients; 1 RCT: PARPi with 136 patients; 1 RCT: TC + PARPi through with 81 patients; 1 RCT: immunotherapy with 149 patients; 1 RCT: TC + immunotherapy at 1.25 mg with 370 patients; 1 RCT: TC + immunotherapy at 2.5 mg with 366 patients; 3 RCTs: chemotherapy with 1,381 patients; 1 RCT: TC + selective small-molecule inhibitor through with 58 patients; 9 RCTs: placebo with 2,375 patients; 9 RCTs: observation with 3,159 patients) reported mature data on OS of OC patients with maintenance therapy. No significant heterogeneity existed in RCTs (overall: *I* ^2^ = 0%; *P* = 0.73), so the pooled HR was calculated by using a fixed-effect model. Except for placebo (HR, 1.02; 95% CI, 0.89–1.16; *P* = 0.76), other treatments significantly improved OS when compared with observation (all *P <*0.05) (**Figure 4B**). Compared with PARP inhibitors indirectly, OS was significantly improved in chemotherapy combined with AIs recurrent (HR, 0.72; 95% CI, 0.53–0.98; *P* = 0.036) (**Figure 5**).

### AEs

Considering the accuracy of the results, we only analyzed the toxicity profiles of placebo maintenance therapy after completing chemotherapy to avoid its effect. Results regarding the patients’ all grades and grade ≥ 3 toxicity profiles were pooled for only 10 placebo maintenance therapy in all included studies. Except for a study with grade ≥ 3 toxicity profiles (45), other RCTs reported all grades and grade ≥ 3 toxicity profiles in detail. Toxicity profiles were classified into total toxicity, hematological toxicities, gastrointestinal toxicities, and other toxicities. In all, 5 studies (9, 14, 41, 42, 46) with 954 patients reported the number of all grades AEs (897 patients) and 6 studies (9, 14, 41, 42, 45, 46) with 994 patients reported the number of grade ≥ 3 AEs (201 patients); the incidences of all grades AEs and grade ≥ 3 AEs were 94.03% (95% CI, 92.53%–95.53%) and 20.22% (95% CI, 17.72%–22.72%), respectively (**Table 2**).

**Table 2:** Overall incidences (%) and 95% confidence intervals of common adverse events in patients with placebo maintenance.

Adverse events	Trials	All grades	Events (*n*)	Total (*n*)	Trials	Grade ≥ 3	Events (*n*)	Total (*n*)
Any	5	94.03	897	954	6	20.22	201	994
		(92.53–95.53)				(17.72–22.72)		
Hematological disorders								
Anemia	5	9.65	81	839	4	1.21	8	660
		(7.65–11.65)				(0.38–2.04)		
Neutropenia	6	6.38	83	1,300	6	1.54	20	1,300
		(5.05–7.71)				(0.87–2.21)		
Thrombocytopenia	5	3.07	36	1,172	5	0.68	7	1,023
		(2.08–4.06)				(0.18–1.18)		
Gastrointestinal disorders								
Nausea	8	26.38	350	1,327	2	1.04	5	482
		(24.01–28.75)				(0.13–1.95)		
Abdominal pain	8	24.34	418	1,717	8	1.44	21	1,463
		(22.31–26.37)				(0.83–2.05)		
Constipation	6	18.92	182	962	3	2.65	10	377
		(16.44–21.40)				(1.03–4.27)		
Diarrhea	8	16.65	257	1,544	5	1.62	16	990
		(14.79–18.51)				(0.83–2.41)		
Vomiting	6	14.55	140	962	6	0.91	8	879
		(12.32–16.78)				(0.28–1.54)		
Other toxicities								
Fatigue	9	29.75	532	1,788	7	1.19	17	1,423
		(27.63–31.87)				(0.63–1.75)		
Hypertension	3	13.89	106	763	3	4.06	31	763
		(11.44–16.34)				(2.66–5.46)		
Headache	9	13.14	235	1,788	3	0.64	5	778
		(11.57–14.71)				(0.08–1.20)		
Insomnia	3	10.29	63	612	1	0.41	1	244
		(7.88–12.7)				[(-0.39) –1.21]		
Dizziness	4	7.06	42	595	1	0.53	1	189
		(5.0–9.12)				[(-0.51) –1.57]		

### Hematological Toxicities

We assessed three common hematological toxicities, including anemia, neutropenia, and thrombocytopenia, in this meta-analysis. The incidences of all grades and grade ≥ 3 toxicities were 9.65% (95% CI, 7.65%–11.65%) and 1.21% (95% CI, 0.38%–2.04%) in anemia, 6.38% (95% CI, 5.05%–7.71%) and 1.54% (95% CI, 0.87%–2.21%) in neutropenia, and 3.07% (95% CI, 2.08%–4.06%) and 0.68% (95% CI, 0.18%–1.18%) in thrombocytopenia, respectively (**Table 2**).

### Gastrointestinal Toxicities

We also assessed several common gastrointestinal toxicities, such as nausea, abdominal pain, constipation, diarrhea, and vomiting. The incidences of all grades and grade ≥ 3 gastrointestinal toxicities were 26.38% (95% CI, 24.01%–28.75%) and 1.04% (95% CI, 0.13%–1.95%) in nausea, 24.34% (95% CI, 22.31%–26.37%) and 1.44% (95% CI, 0.83%–2.05%) in abdominal pain, 18.92% (95% CI, 16.44%–21.4%) and 2.65% (95% CI, 1.03%–4.27%) in constipation, 16.65% (95% CI, 14.79%–18.51%) and 1.62% (95% CI, 0.83%–2.41%) in diarrhea, and 14.55% (95% CI, 12.32%–16.78%) and 0.91% (95% CI, 0.28%–1.54%) in vomiting, respectively (**Table 2**).

### Other Toxicities

Other toxicities like fatigue, hypertension, headache, insomnia, and dizziness were also analyzed in this meta-analysis. The incidences of all grades and grade ≥ 3 toxicities were 29.75% (95% CI, 27.63%–31.87%) and 1.19% (95% CI, 0.63%–1.75%) in fatigue, 13.89% (95% CI, 11.44%–16.34%) and 4.06% (95% CI, 2.66%–5.46%) in hypertension, 13.14% (95% CI, 11.57%–14.71%) and 0.64% (95% CI, 0.08%–1.2%) in headache, 10.29% (95% CI, 7.88%–12.7%) and 0.41% [95% CI, (-0.39%)-1.21%] in insomnia, and 7.06% (95% CI, 5.0%–9.12%) and 0.53% [95% CI, (-0.51%)-1.57%] in dizziness successively (**Table 2**).

## Discussion

Lots of meta-analyses about experimental drugs were performed to estimate the effect on survival. Ours was the first one concentrating on RCTs to assess the placebo effect of maintenance therapy in primary and recurrent OC settings. In this meta-analysis, we proved no statistically significant differences in the survival, whether PFS or OS, of OC patients between placebo and observation (all *P >* 0.05). Until now, no research has focused on this point. The National Comprehensive Cancer Network Guideline believes that participating in clinical trials for any cancer patients is the best management, which is positively encouraged (the corresponding website: nccn.org/clinical trials/member_institutions.aspx.). The ratios of the amount of participants between the experimental and the control arms of the included RCTs were 2:1 (9, 14, 36, 40, 42, 46, 48), 1:1 (1, 5–8, 11–13, 16–19, 32, 34, 38, 39, 41, 43–45, 50–53, 55–57, 58), or 1:1:1 (15, 35, 37, 49, 54). That implied that the participants had the opportunity of 1/3 or 1/2 to take placebo, but our results proved that it did not have an effect on survival. Therefore, it is safe to be ignored when designing patients’ composition in RCTs.

However, placebo produced some AEs—the incidences of all grades and grade ≥ 3 were 94.03% (95% CI, 92.53%–95.53%) and 20.22% (95% CI, 17.72%–22.72%), respectively, which were higher than those of the observation arms and the study of Matías Rodrigo Chacón *et al.* (85.1% in all grades and 18% in grade ≥ 3) (59). The reason of the difference was that our study only included OC patients, while the study of Matías Rodrigo Chacón *et al.* contained cases of melanoma, non-small cell lung cancer, gastrointestinal stromal tumor, and renal cell carcinoma. SOLO_2_ (42), focusing on OC, reported the incidences of placebo-related AEs as 94.95% in all grades and 18.18% in grade ≥ 3, which was similar to our results. Fatigue was the most common AEs, followed by gastrointestinal toxicities. A. Hrobjartsson *et al.* suggested that subjective symptoms, such as pain and anxiety, were affected more easily by placebo effect than objective measures like blood pressure (20). Julia W. Haas *et al.* found that patients with irritable bowel syndrome in a double-blind placebo experiment possessed more enthusiasm. However, those in an open-label placebo research were contradicted and thought that the improvement of symptoms rarely came from the treatment itself but that it was more like a psychological function (25).

The results compared with PARP inhibitors in this study were different from the study of Feng *et al.* (60), the only meta-analysis comparing PARP inhibitors, AIs, and chemotherapy, and showed that PARP inhibitors were superior to AIs and chemotherapy. We considered the following several reasons: (1) our study only included platinum-sensitive OC patients, while Feng’s study included those cases which are platinum-sensitive and platinum-resistant; (2) comprehensive maintenance therapy models were illustrated in this meta-analysis, and the trials’ numbers of immunotherapy were obviously less than those of other treatments, which might affect the weight of data; and (3) Feng’s study merged placebo and observation into one arm, but some results continued to be debatable in the maintenance therapy of RCTs with OC—for example, immunotherapy did not improve the patients’ survival, but PARP inhibitors did so, while in this indirect meta-analysis immunotherapy was prior to PARP inhibitors. We considered that sample sizes and the weight of data produced conflicting results. In the future, a large number of direct comparative clinical trials are needed to confirm the relation between immunotherapy and PARP inhibitors.

Several highlights existed in this meta-analysis, which are as follows: firstly, it was conducted according to PRISMA and included all well-designed and high-quality phase II or phase III RCTs to reduce the risk of bias among trials and increase the reliability of the results. Secondly, it included comprehensive models of maintenance treatment of OC, such as chemotherapy, AIs, PARP inhibitors, immunotherapy, and SSM inhibitors. Thirdly, network meta-analysis was used to indirectly compare the efficacy between placebo and observation and among experimental drugs due to no direct RCTs. Lastly, it firstly stated the incidences of AEs produced by placebo tablets in RCTs with OC and was not only limited to the fatigue in advanced cancer patients (28).

Some limitations were stated in our meta-analysis. First, performing a stratified pooled analysis to reduce the risk of bias among clinical trials according to disease setting (primary *vs*. recurrent OC) was difficult because of the limited clinical trials. Different endpoints existed in the studies, and the number of clinical trials of all kinds of maintenance therapies was less than 10. Second, the data used were based on the clinical trial level rather than the individual patient; data on survival and AEs were not assessed accurately or incorporated into the analysis due to lacking original data which were not available, which were supposed to be more sensitive for toxicity analysis. Third, we only evaluated the incidences of AEs in placebo arms without combination with chemotherapy, which could not better represent all patients included in the RCTs. Lastly, we did not calculate the relative risk ratio of AEs between placebo and observation due to insufficient data on AEs about observation.

## Conclusions

This network meta-analysis indicated that the maintenance therapy of OC improved PFS and partial OS benefits. Compared with observation, placebo did not improve or reduce the PFS or OS benefits, but it increased the incidences of AEs in OC patients. In the future, more clinical trials should be designed to directly confirm the placebo effect.

## Data Availability Statement

The original contributions presented in the study are included in the article/supplementary material. Further inquiries can be directed to the corresponding authors.

## Author Contributions

QL conceived the study idea and wrote the manuscript. JW performed the search in the electronic databases, organized the data, and wrote the manuscript. LH and FC analyzed the data. CS performed the search in the electronic databases and organized the data. YW and XF checked the data again. LZ, YB, KZ, and JJ made modifications in the paper. All authors contributed to the article and approved the submitted version.

## Funding

This work was supported by the Clinical Research Award of the First Affiliated Hospital of Xi’an Jiaotong University, China (XJTU1AF-2018-017 and XJTU1AF-CRF-2019-002), the Natural Science Basic Research Program of Shaanxi (2018JM7073 and 2017ZDJC-11), the Key Research and Development Program of Shaanxi (2017ZDXM-SF-068 and 2019QYPY-138), the Innovation Capability Support Program of Shaanxi (2017XT-026 and 2018XT-002), and the Medical Research Project of Xi’an Social Development Guidance Plan (2017117SF/YX011-3). The funders had no role in the study design, data collection and analysis, decision to publish, or preparation of the manuscript.

## Conflict of Interest

The authors declare that the research was conducted in the absence of any commercial or financial relationships that could be construed as a potential conflict of interest.

## Publisher’s Note

All claims expressed in this article are solely those of the authors and do not necessarily represent those of their affiliated organizations, or those of the publisher, the editors and the reviewers. Any product that may be evaluated in this article, or claim that may be made by its manufacturer, is not guaranteed or endorsed by the publisher.
